# Supplementary material for: Why •CF2H is nucleophilic but •CF3 is electrophilic in reactions with heterocycles
Source: Nat Commun. 2024 May 31;15:4630. doi: 10.1038/s41467-024-48949-z (PMC11143314; doi:10.1038/s41467-024-48949-z)
Supplement: Supplementary file 1 — Supplementary Information [file 41467_2024_48949_MOESM1_ESM.pdf]

Supplementary Information

Why •CF_2H is nucleophilic but •CF_3 is electrophilic in reactions with heterocycles

Meng Duan[1], Qianzhen Shao[1], Qingyang Zhou[1], Phil S. Baran[2], K. N. Houk[1,*]

[1] Department of Chemistry and Biochemistry, University of California, Los Angeles, California 90095, USA

[2] Department of Chemistry, Scripps Research, La Jolla, California 92037, USA.

E-mail: houk@chem.ucla.edu

Table of contents

1. Absolute Calculation Energies, Enthalpies, and Free Energies..2
2. Detailed Methodologies and Expressions for **IP**, χ, ω 4
3. Calculated Gibbs Activation Energy of Unprotonated Transition States 5
4. Generalized Charge Decomposition Analysis..7
5. Restricted Open-shell Computations..10

1. Absolute Calculation Energies, Enthalpies, and Free Energies

Supplementary Table 1 Absolute calculation energies, enthalpies, and free energies at the SMD-M06-2X/def2-QZVPP//SMD-M06-2X/6-311+G(d,p) level of theory.

Geometry	$E_{(solv,M06\text{-}2X)}$[1]	$H_{(solv,M06\text{-}2X)}$[2]	$G_{(solv,M06\text{-}2X)}$[3]	IF[4]
**1a**	-400.958996	0.135448	0.093284	-
**1a-H**	-401.401111	0.149483	0.106580	-
**1b**	-667.506855	0.255097	0.206687	-
**1b-H**	-667.943257	0.268639	0.219671	-
**1c**	-1037.791132	0.458095	0.386125	-
**1c-H**	-1038.240201	0.472102	0.400235	-
**CH_3**	-39.828270	0.033638	0.009745	-
**CH_2F**	-139.085035	0.028782	0.002046	-
**CF_2H**	-238.353314	0.023266	-0.005803	-
**CF_3**	-337.621180	0.016570	-0.013436	-
**TSa-CF_2H-C2**	-639.303986	0.159028	0.106590	-458.50
**TSa-CF_2H-C2-H**	-639.749602	0.173198	0.120421	-392.34
**TSa-CF_2H-C3**	-639.305195	0.159229	0.106709	-449.18
**TSa-CF_2H-C3-H**	-639.748366	0.173353	0.120905	-433.61
**TSa-CF_3-C2**	-738.574252	0.151773	0.097272	-382.76
**TSa-CF_3-C2-H**	-739.015249	0.165824	0.112077	-376.66
**TSa-CF_3-C3**	-738.575419	0.151957	0.097234	-377.32
**TSa-CF_3-C3-H**	-739.014342	0.165913	0.112219	-384.97
**TSb-CF_2H-C2**	-905.853719	0.278849	0.219436	-432.66
**TSb-CF_2H-C2-H**	-906.296451	0.292613	0.233044	-345.85
**TSb-CF_2H-C5**	-905.853927	0.278772	0.218945	-472.88
**TSb-CF_2H-C5-H**	-906.291624	0.292437	0.232741	-489.49
**TSb-CF_3-C2**	-1005.123356	0.271711	0.209916	-370.87
**TSb-CF_3-C2-H**	-1005.560825	0.285737	0.225344	-369.66

**TSb-CF_3-C5**	-1005.125519	0.271600	0.209741	-341.05
**TSb-CF_3-C5-H**	-1005.560804	0.285483	0.224004	-368.49
**TSc-CF_2H-C2**	-1276.137175	0.481933	0.399437	-444.83
**TSc-CF_2H-C2-H**	-1276.590206	0.496031	0.413036	-427.14
**TSc-CF_2H-C7**	-1276.137719	0.482012	0.398434	-464.16
**TSc-CF_2H-C7-H**	-1276.589844	0.496145	0.413397	-450.17
**TSc-CF_3-C2**	-1375.408159	0.474858	0.390400	-364.90
**TSc-CF_3-C2-H**	-1375.856261	0.488875	0.405112	-388.33
**TSc-CF_3-C7**	-1375.408887	0.474753	0.389165	-350.61
**TSc-CF_3-C7-H**	-1375.857566	0.488724	0.404270	-372.47

[1]The electronic energy calculated by M06-2X/def2-QZVPP in dichloromethane solvent. [2]The thermal correction to enthalpy calculated by M06-2X/6-311+G(d,p) in dichloromethane solvent. [3]The thermal correction to Gibbs free energy calculated by M06-2X/6-311+G(d,p) in dichloromethane solvent. [4]The M06-2X calculated imaginary frequencies for the transition states.

2. Detailed Methodologies and Expressions for IP, χ, ω

1. Ionization Potential (IP):

Ionization potential is the energy required to remove an electron from the outermost shell of a molecule. In Density Functional Theory (DFT), it can be calculated using the Supplementary equation (1):

IP = E(cation) - E(neutral)   (1)

where E(cation) represents the energy of the ionized molecule (cationic state), and E(neutral) is the energy of the molecule in its neutral state.

2. Absolute Electronegativity (χ):

Absolute electronegativity represents the tendency of an atom or molecule to attract electrons. In DFT, absolute electronegativity can be calculated using the Mulliken electronegativity concept with the Supplementary equation (2):

χ = (IP+EA)/2   (2)

where IP stands for the Ionization Potential, and EA refers to the Electron Affinity. The Electron Affinity is determined by the formula EA = E(neutral) - E(anion), where E(anion) is the energy of the anionic molecule.

3. Global Electrophilicity (ω):

Global Electrophilicity quantifies the electrophilic nature of a molecule, essentially measuring its ability to accept electrons. In the DFT framework, global electrophilicity can be calculated using the Supplementary equation (3):

$\omega = \mu^2/(2\eta)$ (3)

where μ is the electronic chemical potential, typically approximated as the negative of absolute electronegativity (χ), and η is the chemical hardness, calculated by η = (IP – EA)/2.

3. Calculated Gibbs Activation Energy of Unprotonated Transition States

a)

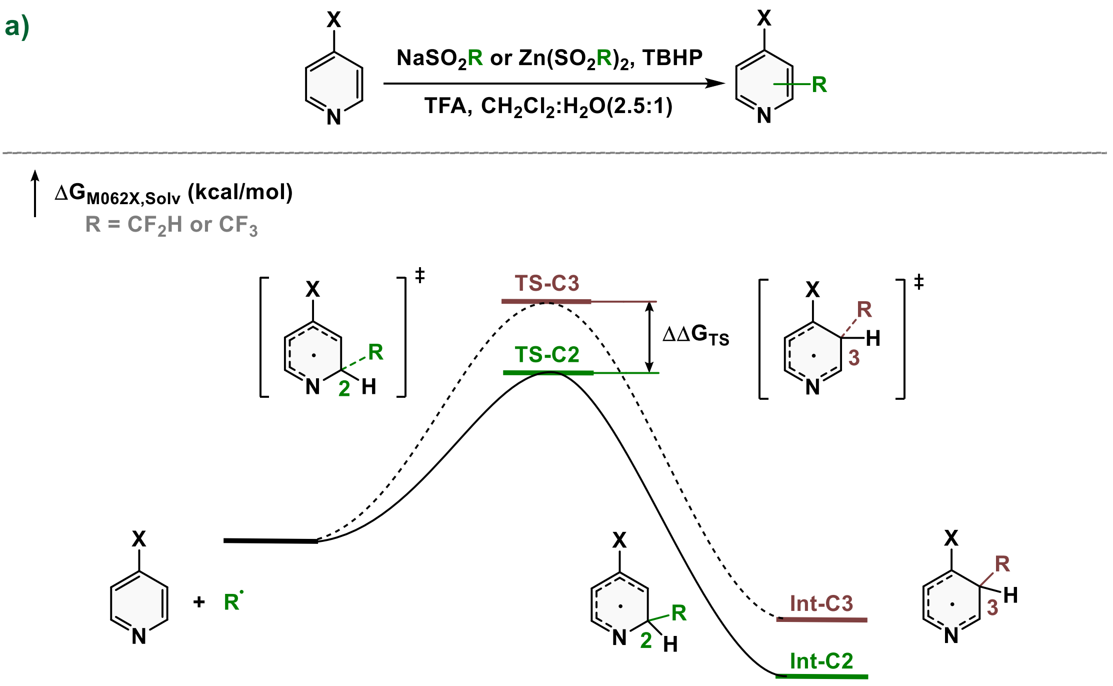

b) $\Delta\Delta G_{TS}$ (Unit: kcal/mol)
(Computed Ratio)
(Experimental Ratio)

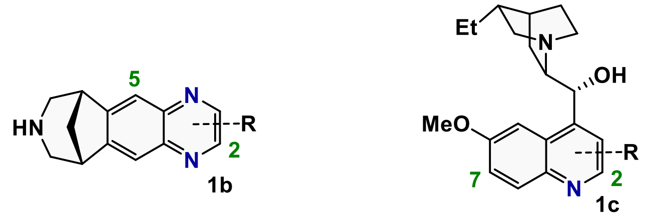

	1a: C2	1a: C3	1b: C2	1b: C5	1c: C2	1c: C7
CF_2H	0.0	-0.7	0.0	-0.4	0.0	-1.0
	(C2 : C3 = 1 : 3.3)		(C2 : C5 = 1 : 2)		(C2 : C7 = 1 : 5.5)	
	(C2 : C3 > 10 : 1)		(C2 only)		(C2 only)	
CF_3	0.0	-0.8	0.0	-1.5	0.0	-1.2
	(C2 : C3 = 1 : 3.9)		(C2 : C5 = 1 : 12.8)		(C2 : C7 = 1 : 7.7)	
	(C2 : C3 = 2.6 : 1)		(C2 : C5 = 1 : 4)		(C7 only)	

Supplementary Fig. 1 a) The energy profile and definition of $\Delta\Delta G_{TS}$. b) Calculated Gibbs activation energy of unprotonated transition states compare to attack on C2, computed ratios (black numbers in parenthesis) and experimental ratios of products (gray numbers in parenthesis) for TSs of radical addition of various heterocycle arenes. Results of experiments are based on Baran's work.

Unprotonated heterocycles were also investigated. For the simple heterocycle 4-acetylpyridine, the calculation shows that both CF_2H and CF_3 radicals prefer the C3 position. In terms of varenicline, both the CF_2H and CF_3 radical additions take place at position C5. In the case of dihydroquinine, the CF_2H and CF_3 radicals both favor the C7 position. As a result, these predicted results are not in agreement with the experimentally observed regioselectivity, suggesting the heterocycle substrates will be protonated under these reaction conditions.

4. Generalized Charge Decomposition Analysis

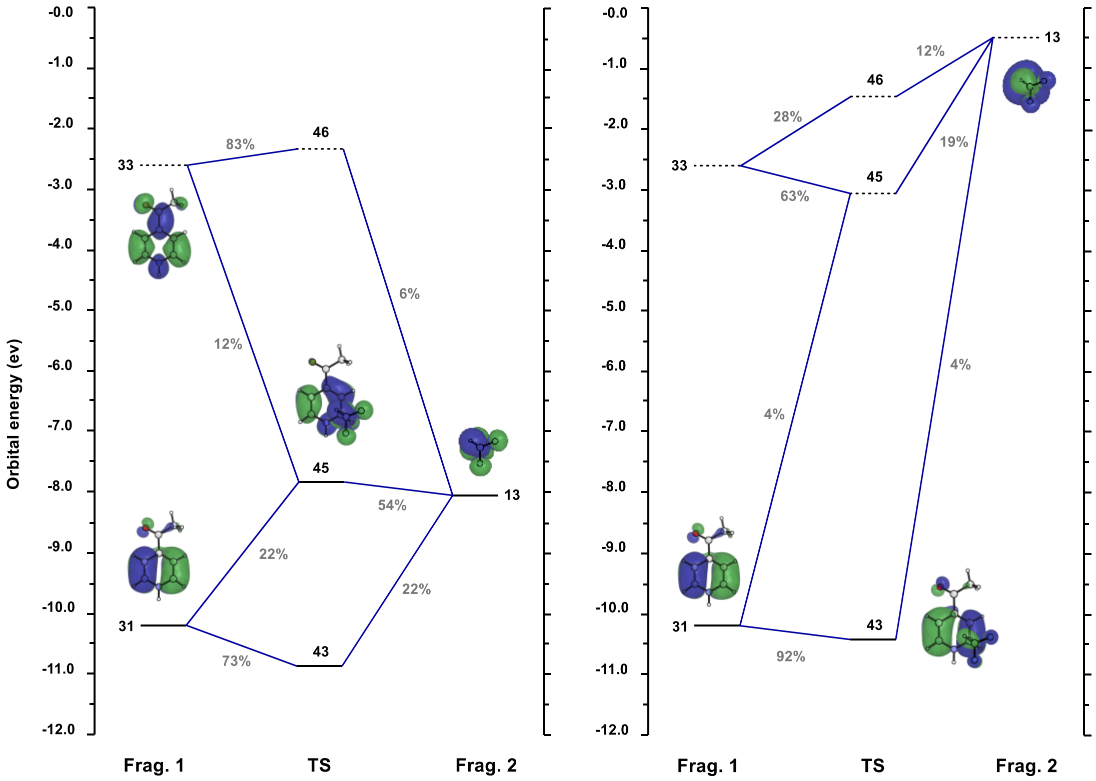

Supplementary Fig. 2 The GCDA plots for alpha and beta orbitals of the CF_2H radical system using **TS-C2** as an example. Energies are in eV. Only important orbitals are shown within the energy range.

The Generalized Charge Decomposition Analysis (GCDA) was also used to study Molecular Orbital (MO) interactions, particularly those involving Frontier MOs. Dotted lines depict empty orbitals. Bold lines represent occupied orbitals. The percentages on the lines correspond to the percentages of the orbitals that are mixed together. In the first place, we examined the TS for CF_2H radical attack on C2 position. For the alpha orbital, the occupied radical SOMO interacts with the pyridine HOMO, leading to a closed-shell interaction. Moreover, the pyridine LUMO could mix with the HOMO and stabilize the TS. This whole interaction demonstrates the nucleophilic properties of the CF_2H radical. As for the beta orbital, the vacant radical SOMO interacts with the pyridine HOMO. Due to the larger energy gap between these two orbitals, this interaction is weaker compared to the α part. Thus, LUMO stabilization plays a crucial role in CF_2H radical addition, and the LUMO coefficient dominates regioselectivity.

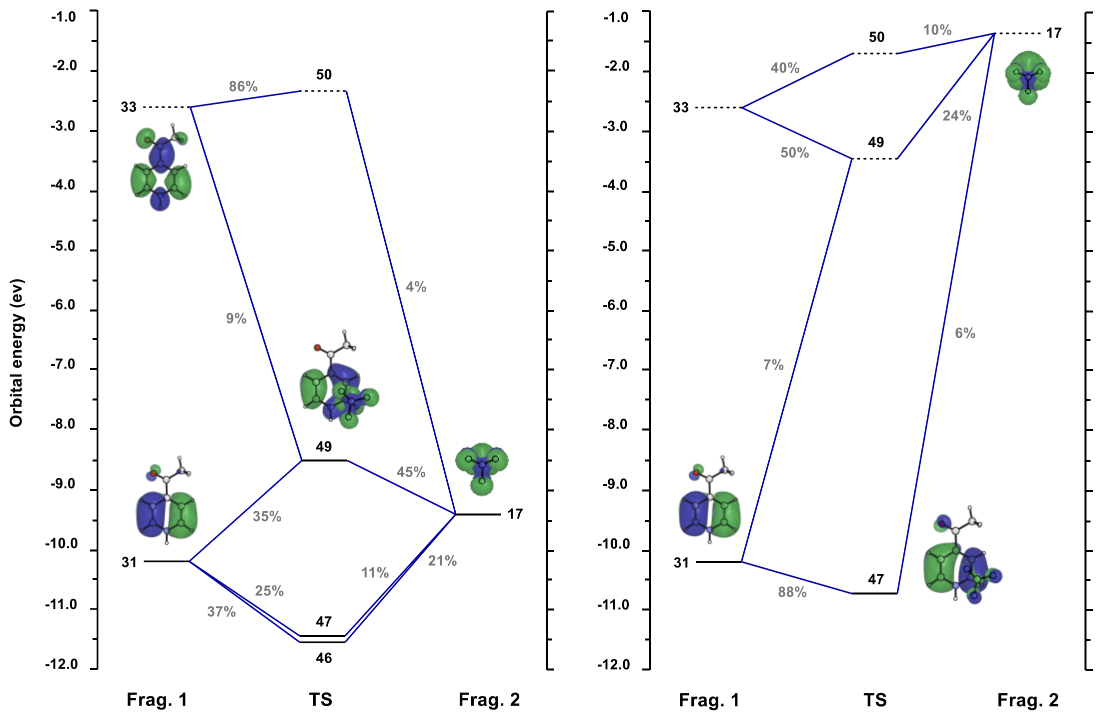

Supplementary Fig. 3 The GCDA plots for alpha and beta orbitals of the CF_3 radical system using **TS-C2** as an example. Energies are in eV. Only important orbitals are shown within the energy range.

We next explored the TS for CF_3 radical attack on C2 position. The GCDA illustrates the key differences between CF_3 and CF_2H radicals. For the alpha part, the occupied CF_3 radical SOMO has a significantly lower orbital energy, resulting in less interaction with the pyridine LUMO. Therefore, pyridine LUMO will cause less positional differentiation. For the beta part, the lower vacant radical SOMO increases the SOMO-HOMO interaction. This greater interaction reflects the electrophilic character of the CF_3 radical in comparison to the CF_2H radical. Due to this, both HOMO and LUMO coefficients play a significant role in determining the regioselectivity of the CF_3 radical addition.

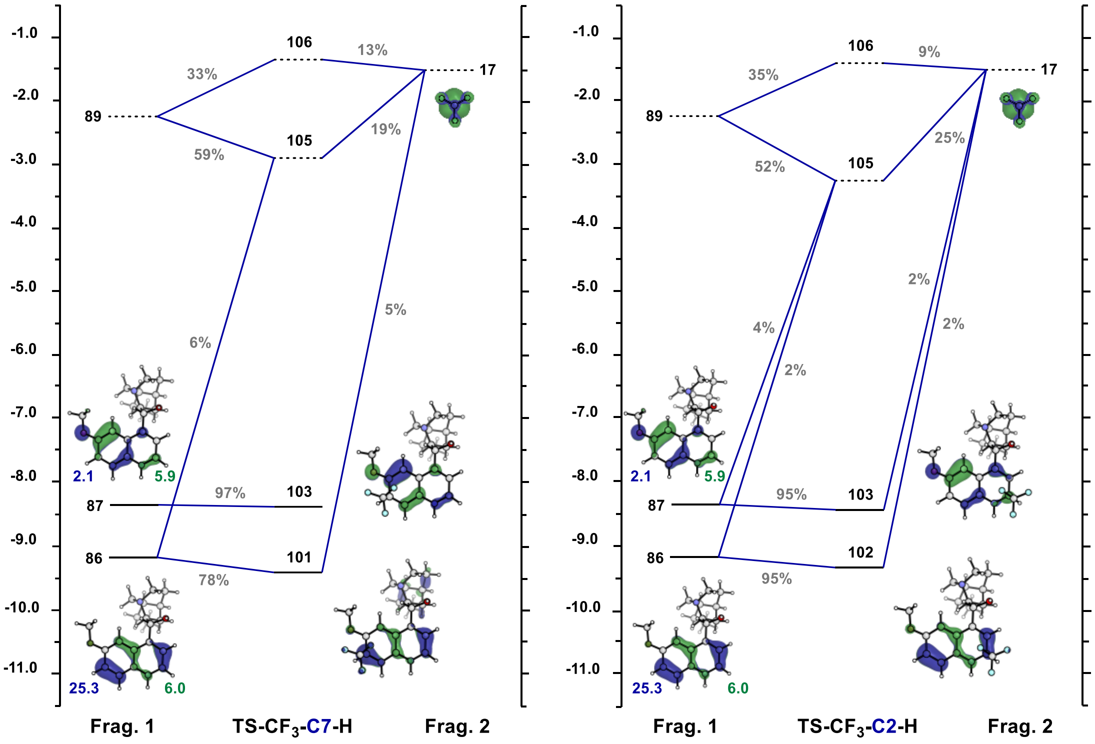

Supplementary Fig. 4 The GCDA plots for beta orbitals of **TS-C7** and **TS-C2**. Energies are in eV. Only important orbitals are shown within the energy range.

We investigated the TSs for CF_3 radical attack on C7 and C2 positions. For the C7 attack, a node on C7 of the dihydroquinine HOMO makes the vacant radical SOMO-β interacts with the HOMO-1 rather than HOMO. Due to this, the HOMO-1 coefficient plays an important role in determining regioselectivity. For the C2 attack, the coefficients of HOMO and HOMO-1 on the C2 position are almost identical (5.9 and 6.0), leading to similar interactions with the vacant radical SOMO-β. However, the coefficient on C2 is quite small in comparison to the coefficient on C7 (25.3). As a result, C7 is preferred over C2 for dihydroquinine.

5. Restricted Open-shell Computations

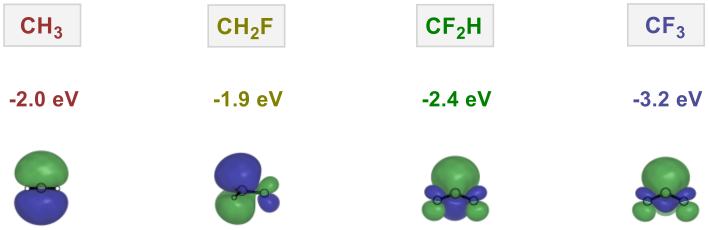

Supplementary Fig. 5 Side views of radical SOMO and restricted open-shell orbital energies of different radicals using the Hartree-Fock (HF) method.

The Hartree-Fock (HF) method has now been applied to orbitals. As noted by many experts, the general shapes of HF and KS orbitals, as well as relative energies for a similar series produce the same type of understanding that we have sought in this paper.

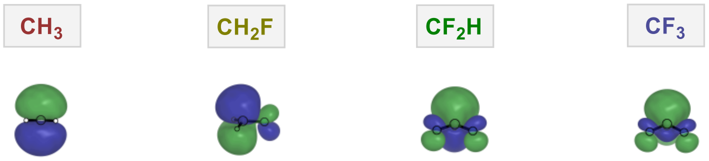

Supplementary Fig. 6 Side views of natural orbitals using the CCSD method.

Additionally, we employed the CCSD method to obtain the natural orbitals. The natural orbitals are similar to the Kohn-Sham DFT orbitals. However, it's important to note that the energies of these natural orbitals are not physically meaningful since they are not the eigenfunction of Fock operator.
